# Accuracy of a Dual Path Platform (DPP) Assay for the Rapid Point-of-Care Diagnosis of Human Leptospirosis

**DOI:** 10.1371/journal.pntd.0001878

**Published:** 2012-11-01

**Authors:** Scott A. Nabity, Guilherme S. Ribeiro, Carolina Lessa Aquino, Daniele Takahashi, Alcinéia Oliveira Damião, André H. O. Gonçalves, Demócrito B. Miranda-Filho, Rena Greenwald, Javan Esfandiari, Konstantin P. Lyashchenko, Mitermayer G. Reis, Marco A. Medeiros, Albert I. Ko

**Affiliations:** 1 Duke University School of Medicine, Durham, North Carolina, United States of America; 2 Centro de Pesquisa de Gonçalo Moniz, Fundação Oswaldo Cruz, Salvador, Brazil; 3 Instituto de Saúde Coletiva, Universidade Federal da Bahia, Salvador, Brazil; 4 Bio-Manguinhos, Fundação Oswaldo Cruz, Rio de Janeiro, Brazil; 5 Departamento de Medicina Clínica, Universidade de Pernambuco, Recife, Brazil; 6 Chembio Diagnostic Systems, Medford, New York, United States of America; 7 Yale University Schools of Public Health and Medicine, New Haven, Connecticut, United States of America; University of Washington, United States of America

## Abstract

**Background:**

Diagnosis of leptospirosis by the gold standard serologic assay, the microscopic agglutination test (MAT), requires paired sera and is not widely available. We developed a rapid assay using immunodominant *Leptospira* immunoglobulin-like (Lig) proteins in a Dual Path Platform (DPP). This study aimed to evaluate the assay's diagnostic performance in the setting of urban transmission.

**Methodology:**

We determined test sensitivity using 446 acute and convalescent sera from MAT-confirmed case-patients with severe or mild leptospirosis in Brazil. We assessed test specificity using 677 sera from the following groups: healthy residents of a Brazilian slum with endemic transmission, febrile outpatients from the same slum, healthy blood donors, and patients with dengue, hepatitis A, and syphilis. Three operators independently interpreted visual results without knowing specimen status.

**Results:**

The overall sensitivity for paired sera was 100% and 73% for severe and mild disease, respectively. In the acute phase, the assay achieved a sensitivity of 85% and 64% for severe and mild leptospirosis, respectively. Within seven days of illness onset, the assay achieved a sensitivity of 77% for severe disease and 60% for mild leptospirosis. Sensitivity of the DPP assay was similar to that for IgM-ELISA and increased with both duration of symptoms (chi-square regression P = 0.002) and agglutinating titer (Spearman ρ = 0.24, *P*<0.001). Specificity was ≥93% for dengue, hepatitis A, syphilis, febrile outpatients, and blood donors, while it was 86% for healthy slum residents. Inter-operator agreement ranged from very good to excellent (kappa: 0.82–0.94) and test-to-test reproducibility was also high (kappa: 0.89).

**Conclusions:**

The DPP assay performed acceptably well for diagnosis of severe acute clinical leptospirosis and can be easily implemented in hospitals and health posts where leptospirosis is a major public health problem. However, test accuracy may need improvement for mild disease and early stage leptospirosis, particularly in regions with high transmission.

## Author Summary

Leptospirosis is an important cause of acute fever in the tropics and the mortality rate may exceed 15% in patients with severe disease manifestations. The gold standard serological test for diagnosing leptospirosis, the microagglutination test or MAT, requires significant laboratory resources and results are not timely. Improved diagnostics are therefore critically needed to identify patients and outbreaks earlier and to thereby prevent unnecessary deaths. The need for a rapid diagnostic test is particularly acute in resource-poor settings where leptospirosis is a major public health problem and sophisticated laboratories are unavailable. In this study, we measured the diagnostic accuracy of the novel Dual Path Platform (DPP) for leptospirosis using serum from patients with mild and severe disease. The DPP assay detected up to 85% of severe leptospirosis and 64% of mild leptospirosis patients using the initial clinical specimen collected at hospital presentation and its diagnostic performance was comparable to a commonly used IgM-ELISA. Furthermore, the DPP assay produces a result in 20 minutes and can be more easily implemented in field settings than existing diagnostic technologies. The commercially available DPP kit offers the simple, accurate, and quick diagnosis of leptospirosis and, consequently, more timely clinical and public health decision-making.

## Introduction

Leptospirosis, caused by >200 pathogenic serovars of *Leptospira interrogans*, is an increasingly important cause of morbidity worldwide with >500,000 cases annually [1], [2]. Most urban infections in Brazil and other emerging economy countries occur in densely populated, resource-poor slums that lack adequate sanitation, cultivate rodent reservoirs, and foster the environmental persistence of *Leptospira* 3–[6]. Although few (5–10%) infections progress to severe disease, typified by jaundice, acute renal failure, and hemorrhage (Weil's disease) and/or respiratory compromise, the case fatality may exceed 15% when severe disease develops [7], [8]. Early antimicrobial therapy reduces illness duration and severity [9], [10]. However, leptospirosis is often clinically confused with other acute febrile illnesses [11], [12]. Accurate early detection therefore remains urgently needed to avert the significant consequences of leptospirosis.

Culturing *Leptospira* is difficult and growth success is diminished in patients already initiated on antimicrobial therapy. The gold standard diagnostic assay for leptospirosis, the microscopic agglutination test (MAT), requires skilled technicians, maintenance of live cultures, and paired sera for confirmation. Application of these standard confirmatory techniques is limited and prolonged [13], [14], thus hindering patient management, community-based surveillance, and outbreak response. Polymerase chain reaction (PCR) is ≤60% sensitive in the acute phase and is consistently outperformed by serological tests [15], [16]. Current PCR and enzyme-linked immunoassay (ELISA) systems further require sophisticated equipment. Agglutination, dipstick, and lateral flow assays are among other diagnostic technologies for leptospirosis whose performance has been described [17]–[24]. Collectively, these assays demonstrated insufficient sensitivity in early acute disease and some require basic laboratory support.

Most rapid serological tests to date relied on genus-wide cross-reactivity to detect antigenically diverse pathogens, most commonly utilizing whole-cell antigen from the saprophytic serovar Patoc I [7]. The novel Dual Path Platform (DPP) (Chembio Diagnostic Systems, Medford, New York, USA) assay for leptospirosis incorporates high concentrations of recombinant leptospiral immunoglobulin-like (rLig) proteins as antigens. It thereby avoids the cross-reactivity observed in whole-cell assays with nonspecific cell surface components, such as lipopolysaccharides, that are common to other pathogens. Lig proteins are key markers for the serodiagnosis of acute-phase leptospirosis because they elicit a robust humoral immune response [25], [26], are conserved among pathogenic species [27], [28], and are active in natural infection as they are preferentially expressed at physiological osmolarity [29]–[31] and contribute to cell adhesion [32]–[34]. We rationally selected the most seroreactive combination of rLig proteins for use as antigens in the DPP assay for leptospirosis using a multi-antigen print immunoassay (MAPIA) (unpublished data). The DPP has been successfully applied to the diagnosis of other human diseases, including syphilis [35], and utilizes a variation of lateral flow technology, whereby the biological sample and the colorimetric marker are delivered on separate, perpendicular nitrocellulose membranes. This design increases assay sensitivity by circumventing non-specific interference between the assay's embedded marker proteins and immunoglobulin in the patient sample.

In this study, we assessed the diagnostic performance of the DPP assay in the setting of urban leptospirosis transmission using the MAT as the gold standard to determine the primary outcomes of sensitivity, specificity, and reproducibility. Secondarily, we compared its diagnostic accuracy with a commonly used IgM-ELISA and correlated DPP performance with severity and duration of illness.

## Methods

### Ethics statement

We adhered to comprehensive diagnostic accuracy evaluation standards (Table S1) [36] and received IRB approval from FIOCRUZ, New York Presbyterian Hospital, and Yale University. Leptospirosis case-patients, non-leptospirosis febrile outpatients and healthy slum residents provided written consent and blood donors consented to its use in biomedical research. We procured sera for hepatitis A, dengue, and syphilis as anonymous reference specimens.

### Participants

We measured sensitivity using 446 serum samples from 378 individuals with either mild or severe leptospirosis from two urban Brazilian populations. We collected acute sera at enrollment and convalescent samples after approximately 15 days. Case-patient sera from all sites were well characterized according to clinical presentation, clinical and diagnostic laboratory results, epidemiological risk factors, and clinical outcomes using standardized data collection tools based on active case detection protocols [37]. We designated hospitalized case-patients as having severe leptospirosis, regardless of clinical syndrome, and non-hospitalized case-patients as mild leptospirosis. Both mild and severe leptospirosis case-patients were included solely on the basis of serological confirmation by the following MAT criteria: i) seroconversion (undetectable acute titer and convalescent titer ≥1∶200), ii) ≥four-fold rise in acute to convalescent titers, or iii) single sample titer ≥1∶800. We calculated specificity from 677 control sera.

#### Severe disease from Salvador

We randomly selected 259 (18%) of 1,435 acute and 110 (11%) of 1,026 convalescent sera from a serum bank of hospitalized case-patients ≥5 years of age with confirmed leptospirosis. Acute and convalescent specimens were independently sampled and resultant individual case-patient serum pairs were matched thereafter. The serum bank was created between 1996–2010 while conducting active surveillance at the state reference infectious disease hospital in Salvador, Brazil (4.0 cases per 100,000 residents in 2009 [38]), which receives 90% of the region's severe cases. Active surveillance inclusion criteria were: i) strong clinical suspicion for leptospirosis or ii) at least one of the following: acute undifferentiated fever associated with either bleeding, acute renal insufficiency, jaundice, or acute liver injury with transaminases <1,000 U/L.

#### Severe disease from Recife

We also included acute-phase sera from all 23 confirmed case-patients that we recruited at a teaching hospital from June–August 2010 in Recife, Brazil (4.7 per 100,000 in 2009 [38]) using the same active surveillance inclusion criteria. The hospital is one of the two reference centers in Recife for the management of leptospirosis and it reports about 40% of the city's severe cases. Only five of these 23 cases had convalescent sera available. While we used these convalescent specimens for confirming patient status by MAT, we did not include this small group of samples in testing with the DPP assay.

#### Mild disease

Mild cases were identified during community-based sentinel surveillance for acute febrile illness requiring medical attention in the only public outpatient emergency unit serving the community of Pau da Lima within the city of Salvador. As we previously reported, this slum community lacks sanitation infrastructure and is endemic for leptospirosis; 15% of its inhabitants have agglutinating antibodies to *Leptospira* [5]. From 2009–2010, the surveillance team identified 12,198 community residents ≥5 years of age seeking care due to an acute febrile illness. Of these, 23% were recruited and their sera tested for leptospirosis. Available sera (28 acute-phase and 26 convalescent-phase samples) from all 28 mild case-patients with confirmed leptospirosis identified during surveillance were included in this evaluation.

#### Controls

Healthy control sera were derived from: 1) 162 randomly selected Salvador slum residents from a Pau da Lima community survey in 2003–2004, 2) 150 Salvador blood donors, and 3) 100 blood donors from the U.S. where leptospirosis is rare [39]. We selected the aforementioned samples without regard for MAT status. We also included 65 sera each from cases of dengue confirmed by IgM-ELISA or NS1 antigen detection assays and acute hepatitis A confirmed with an IgM chemiluminescent assay at the state reference laboratory in Salvador. Both diseases are in the differential diagnosis for acute clinical leptospirosis. We additionally included 70 acute-phase sera from febrile outpatients randomly selected from the same serum bank used for selecting mild cases, which was created as previously described during slum community-based surveillance for acute febrile illness in 2009–2010. All 70 cases had negative results by MAT (titer <1∶100) for both acute and convalescent sera. Finally, we tested 65 sera from syphilis cases confirmed by VDRL at the state reference laboratory to assess for cross-reacting antibodies [40].

### Laboratory procedures

#### IgM-ELISA and MAT

We assayed sera from all case-patients, healthy slum residents, and Brazilian blood donors using whole-*Leptospira* IgM-ELISA at the Oswaldo Cruz Foundation (FIOCRUZ, Salvador, Brazil) according to manufacturer instructions (Bio-Manguinhos, Rio de Janeiro, Brazil [41]; or PanBio Ltd., Brisbane, Australia [42]). We performed the MAT at the leptospirosis reference laboratory at FIOCRUZ as previously described [14]. Table S2 displays the strains of *Leptospira* used for the MAT. For severe leptospirosis from Salvador, we used a panel of 10 reference and locally isolated strains representing nine serovars and nine serogroups. This panel effectively identified most locally circulating *Leptospira*, 90% of which are *L. interrogans* Icterohaemorrhagiae serovar *copenhageni* [37]. For mild disease specimens, we screened sera from acute febrile illness patients for leptospirosis infection with an IgM-ELISA [41] and with an MAT panel of seven strains representing five serovars and six serogroups. We then applied an extended battery of 26 strains (23 serogroups and 25 serovars) if the sera were IgM-ELISA positive or reacted with any strain in the initial MAT panel with a titer ≥1∶200. For Recife specimens, we used the extended battery of 26 strains. We tested a sample of 53% of control sera from healthy slum residents using a reduced MAT panel of seven strains.

#### Dual Path Platform (DPP) assay

The DPP assay for leptospirosis was developed by Chembio Diagnostic Systems (Medford, New York, USA) and is manufactured by Bio-Manguinhos (Rio de Janeiro, Brazil) through a technology transfer agreement with the Brazilian Ministry of Health. We assayed the DPP at FIOCRUZ (92.5% of all assays) or Chembio Diagnostic Systems (7.5% of all assays) laboratories from March–June 2011 with the same stored sera as were used for IgM-ELISA and MAT. We performed the assay according to manufacturer instructions using 5 µl of undiluted serum. Three independent operators visually interpreted results after 20 minutes as either positive or negative. We defined the final assay result according to the equivalent subjective visual interpretations (i.e., positive or negative) of ≥2 of the 3 operators. Weak reactions were classified according to the same criterion. Interpreters were blinded to case-patient status.

### Statistical analyses

We ordered all samples in random sequence and assigned a blinded unique numerical code prior to testing. We double entered and cross-validated all data elements and analyzed the data with SAS v9.2 (SAS Inst.; Cary, NC, USA) using α = 0.05.

#### Comparison across case-patient groups

We compared demographic, clinical, and laboratory characteristics across case-patient sampling groups using frequencies and means with standard deviations. We used the chi-square test (with continuity correction when ≥1 cell had an expected count <5 units) for comparisons involving categorical variables, and the ANOVA test for comparisons of continuous variables. We presumed the infecting serogroup to be that with the highest MAT titer among both acute and convalescent samples.

#### DPP sensitivity and specificity

We calculated the overall sensitivity for case-patients with paired sera whereby a reactive DPP result in either the acute or convalescent sample was counted as positive. We further reported sensitivity independently for acute and convalescent samples and defined specificity separately within control groups. Exact 95% confidence limits were calculated for sensitivities and specificities, and DPP performance was compared to IgM-ELISA and the geometric mean reciprocal MAT titer. Lastly, we performed chi-square regression by prevalence trend analysis of DPP sensitivity as a function of days of illness [43].

#### DPP predictive values

We estimated the positive predictive value (PPV) and negative predictive value (NPV) of the DPP for severe disease based on empirical data from ongoing active surveillance in Salvador, where pre-test probability was 90% among hospitalized cases suspected of severe leptospirosis meeting surveillance inclusion criteria [37]. Because the prevalence of mild leptospirosis was 1% in the indiscriminate febrile outpatient population (unpublished data), we estimated PPV and NPV for mild disease using a 50% pre-test probability of leptospirosis. We believe this more plausibly reflects the clinical scenario under which physicians will solicit rapid testing for leptospirosis in the outpatient setting. By logistic regression, we assessed whether severe disease clinical characteristics generally known at initial evaluation were able to predict DPP positivity, assessed global model diagnostics, and calculated odds ratios (OR).

#### Reproducibility

We interpreted inter-operator reproducibility (the pairwise concordance of the dichotomized visual result for the same assay) among three operators and test-to-test reproducibility (the concordance of the dichotomized visual result for repeated assay of the same sample) for the DPP assay with kappa. For test-to-test reproducibility, we randomly selected 98 (9%) of the 1,123 originally assayed sera.

## Results

### Case-patient characteristics

Severe disease case-patients providing acute-phase sera from Salvador were older and more frequently male than those with mild disease, whereas demographics between severe disease groups were similar (Table S3). In comparison to those providing acute-phase sera (Table S3), the 110 severe leptospirosis cases-patients from Salvador providing convalescent-phase sera less frequently died (0%; chi-square *P*<0.001).

Acute sera for mild case-patients were collected earlier than for severe disease (Table S3); mild disease sera were collected within two days of symptoms onset for 70% compared to <4% for severe disease from Salvador (chi-square P<0.001). Case-patients designated as mild leptospirosis had objectively less severe disease than those designated as severe leptospirosis per several clinical indicators (Table S3), which correlated with mild disease less frequently diagnosed clinically as leptospirosis (95% for severe vs. 7% for mild; chi-square P<0.001). Among those with severe disease, Salvador case-patients were sicker according to clinical jaundice, oliguria, tachypnea, elevated serum creatinine (≥4 mg/dL), and total serum bilirubin (>1.5 mg/dL) (Table S3).

Most case-patients from Salvador (96% of severe and 93% of mild) had infections presumptively caused by the locally dominant serogroup, *L. interrogans* Icterohaemorrhagiae, compared with 70% from Recife (chi-square P<0.001). Finally, case-patient groups differed by MAT confirmation criteria. Few Recife case-patients had convalescent specimens available and consequently a significantly greater proportion was confirmed with a single titer ≥1∶800 (Table S3).

### Sensitivity and specificity

The overall sensitivity for the 42 severe disease and 26 mild disease patients with paired sera evaluated by DPP was 100% (95% CI 92–100%) and 73% (52–88%), respectively. Sensitivity did not differ significantly between laboratories (data not shown). We measured higher DPP sensitivity in the acute phase for severe disease from Salvador (85%) and Recife (78%) compared to mild disease (64%) (Table 1). Sensitivity was lower for sera collected <7 days after disease onset: 77% for severe disease from Salvador, 43% from Recife, and 60% for mild disease. For severe case-patients from Salvador collected <7 days of onset, the acute-phase sensitivity for DPP (77%, 66–85%) was superior to the 1:00 MAT screening titer (46%, 35–58%; P<0.001) and showed a trend toward superiority over the IgM-ELISA (65%, 54–76%; P = 0.12). In convalescence, the sensitivity was 98% for severe disease from Salvador and 50% for mild disease. Of 18 DPP-positive mild acute sera, seven (39%) were negative in convalescence, despite an increase in MAT titer from the acute phase for six (data not shown).

**Table 1:** Sensitivity of the DPP assay and IgM-ELISA for diagnosing human leptospirosis.

					Sensitivity % (95% confidence interval)	
Case group	Source city	Disease phase		N	DPP	IgM-ELISA
Severe cases	Salvador*	Acute	All acute	259	85 (80–89)	82 (76–86)
			<7 days after onset	81	77 (66–85)	65 (54–76)
			≥7 days after onset	178	89 (84–94)	89 (83–93)
		Convalescent		110	98 (94–100)	99 (95–100)
	Recife†	Acute	All acute	23	78 (56–93)	91 (72–99)
			<7 days after onset	7	43 (10–82)	86 (42–100)
			≥7 days after onset	16	94 (70–100)	94 (70–100)
Mild cases	Salvador‡	Acute	All acute	28	64 (44–81)	57 (37–76)
			<7 days after onset	25	60 (39–79)	52 (31–72)
			≥7 days after onset	3	100 (29–100)	100 (29–100)
		Convalescent		26	50 (30–70)	54 (33–73)

**NOTE.** DPP = Dual Path Platform assay; ELISA = enzyme-linked immunosorbent assay.

* Random samples of acute and convalescent specimens from severe disease case-patients from Salvador were selected independently, after which 42 serum pairs from individual case-patients were identified.

† Represents 5 case-patients with paired sera as convalescent samples from severe cases from Recife were generally not available for testing.

‡ Represents 26 mild disease case-patients with paired sera.

DPP specificity was >95% except among Brazilian blood donors (93%) and slum residents (86%), for which IgM-ELISA outperformed DPP (chi-square P = 0.001) (Table 2). Among the 86 slum residents for whom MAT titers were known, DPP specificity (87%, 78–93%) was also inferior to the MAT screening titer 1∶100 (97%, 90–99%; P = 0.05); rather, it was equivalent to the titer 1∶50 (86%, 77–93%).

**Table 2:** Specificity of the DPP assay and IgM-ELISA for diagnosing human leptospirosis.

		Specificity % (95% confidence interval)	
Control group	N	DPP	IgM-ELISA
Outpatients with fever* †	70	99 (92–100)	99 (92–100)
Dengue cases*	65	100 (95–100)	ND
Hepatitis A cases*	65	95 (87–99)	ND
Syphilis cases*	65	95 (87–99)	ND
Healthy slum residents*	162	86 (80–91)	97 (93–99)
Healthy blood donors from Brazil*	150	93 (87–96)	97 (93–99)
Healthy blood donors from the United States	100	98 (93–100)	ND

**NOTE.** DPP = Dual Path Platform; ELISA = enzyme-linked immunosorbent assay; ND = not determined.

* Samples obtained from individuals in Salvador, Brazil.

† Outpatients with acute fever (microagglutination titer <1∶100 in both acute and convalescent samples).

### Factors influencing DPP sensitivity

**Figure 1:**
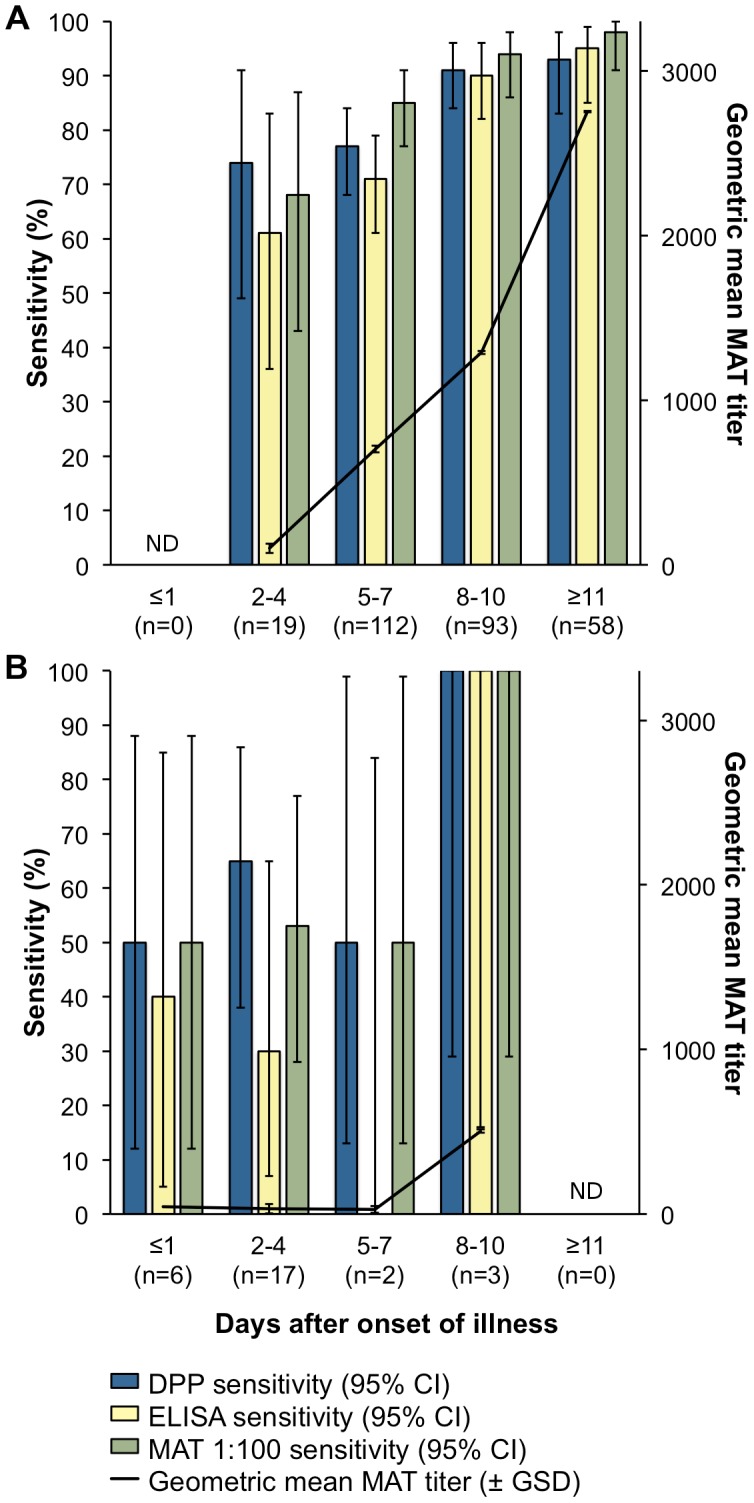
DPP, IgM-ELISA, and 1∶100 MAT sensitivity and mean MAT titer for acute-phase sera of human leptospirosis. Acute-phase sera from (A) 282 MAT-confirmed severe leptospirosis case-patients (259 from Salvador and 23 from Recife, Brazil) and from (B) 28 MAT-confirmed mild leptospirosis case-patients from Salvador. 95% confidence intervals calculated for point estimates of sensitivity and the geometric mean (± geometric standard deviation) calculated for reciprocal MAT titers. DPP = Dual Path Platform; MAT = microagglutination test; ELISA = enzyme-linked immunosorbant assay; ND = not determined because no case-patients were identified within the corresponding time interval.

Sensitivity for both DPP and IgM-ELISA was positively correlated with duration of symptoms in both severe disease from Salvador and Recife (combined in Figure 1A) and mild disease (Figure 1B). In the 14 days after onset for severe leptospirosis, regression on prevalence analysis (chi-square = 10.1, P = 0.002) estimated a daily increase in DPP sensitivity of 2.7%. Notably, the DPP assay outperformed IgM-ELISA early in both severe and mild disease, when treatment initiation is critical. We similarly found a positive relationship between symptom duration and MAT titer for the combined cases of severe disease from Salvador and Recife, and for the mild disease cases (Spearman ρ = 0.24, *P*<0.001). The proportion of all severe disease acute specimens with high MAT titers (≥1∶800) was 17% on days 2–3 after onset and then rose to 98% after day 11 (data not shown).

Severe leptospirosis case-patients with more serious clinical manifestations had an increased likelihood of a positive DPP result (data not shown). Sensitivity varied according to higher serum creatinine (91% for creatinine ≥4 mg/dL vs. 79% for creatinine <4 mg/dL, chi-square P = 0.03) and clinical jaundice (88% for jaundiced vs. 60% for not jaundiced, chi-square P<0.001), but we found no difference by presumptive infecting serogroup (85% for each Icterohaemorrhagiae and other serogroups; chi-square P = 0.95). A logistic regression model incorporating days of illness (OR 1.25, 95% CI 1.06–1.47), jaundice (OR 2.94, 95% CI 1.10–7.84), and serum creatinine ≥4 mg/dL (OR 1.24, 95% CI 1.01–1.54) (global Wald chi-square = 19.1, P<0.001) suggested that duration of illness and disease severity independently influenced DPP performance.

Based on the pre-test probability of 90%, we estimated PPV and NPV of the DPP assay for severe acute clinical leptospirosis to be 98% and 39%, respectively. Using the pre-test probability of 50% for mild disease in the outpatient setting, the estimated PPV and NPV were 81% and 69%, respectively.

### Reproducibility

**Figure 2:**
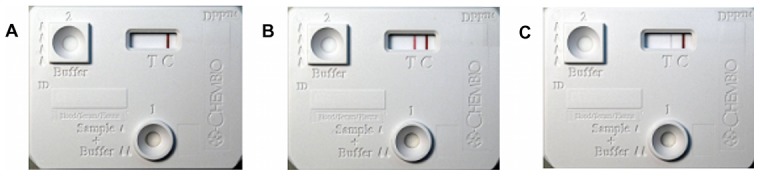
Representative non-reactive, strongly reactive, and weakly reactive DPP assay results. Demonstration of (A) non-reactive, (B) strongly reactive, and (C) weakly reactive visual interpretations for the DPP assay. Coloration of the test line (“T” on rapid test cartridge) indicates the presence of anti-rLig antibodies in the biological sample while coloration of the control line (“C” on rapid test cartridge) indicates the presence of non-specific antibodies and denotes a valid test result. DPP = Dual Path Platform.

Inter-operator reproducibility across three operators was very good to excellent (kappa 0.82, 95% CI 0.76–0.89 to kappa 0.94, 95% CI 0.92–0.96) and test-to-test reproducibility was very good (kappa 0.89, 0.80–0.98). Upon repeat testing, 92% of originally positive and 97% of originally negative assays were interpreted concordantly. Diagnostic performance correlated with the intensity of the assay's reaction. When no colored band was visualized at the test line (Figure 2A), the probability of a properly assigned negative result was 89–91%. Similarly, the probability of correctly identified case-patient status was 95–100% for assays with moderate to strong reactivity (Figure 2B). The probability of an accurate classification was lower, however, with weakly reactive (Figure 2C) interpretations (71–83%).

## Discussion

We measured the diagnostic performance of a novel point-of-care immunoassay for leptospirosis developed from rLig protein fragments. The DPP assay, which detects both IgM and IgG, is sensitive for acute-phase severe leptospirosis and was superior to IgM-ELISA in the first week of illness. We previously reported the superior immunoblot IgM detection against recombinant Lig proteins compared with whole-cell IgM-ELISA and other recombinant proteins [25]. The DPP assay further improves on existing technology by independently delivering the biological sample and the antibody-detecting conjugates to the test line. This method thereby reduces interference between the immunoglobulins in the biological sample and their conjugate proteins that may occur with conventional, single path lateral-flow assays.

Lower sensitivity for mild illness was observed for both DPP and IgM-ELISA, perhaps due to earlier patient presentation in the outpatient setting when immunoglobulin development is underway. Alternatively, an altogether weaker antibody response to mild leptospirosis may occur [44]. Similarly low sensitivity in mild disease convalescence was noted for other rapid serological tests [45] and in our previous work using an rLig membrane-based assay among febrile outpatients from Thailand. Like mild case-patients in the present study, we found that those from Thailand did not require hospitalization, presented earlier, and had lower MAT titers (unpublished data). Lastly, mild case-patients had nondistinctive clinical presentations, were more frequently confirmed with a single MAT titer ≥1∶800, and resided in a high-risk area for previous exposures. Some of the mild case-patients included in this study therefore may have presented with other diseases erroneously attributed to acute clinical leptospirosis.

The sensitivities for severe leptospirosis from Salvador (85%) and Recife (78%) were not statistically different, a comparison limited in power by the small sample of confirmed case-patients from Recife. However the trend toward lower sensitivity in Recife may be explained by disease severity. Both recruitment sites for severe leptospirosis used the same inclusion criteria and both serve as state reference hospitals for severe leptospirosis, yet severe leptospirosis case-patients from Recife were less acutely ill than those from Salvador.

The DPP assay specificity was good in both sick (i.e., illnesses other than leptospirosis) and healthy populations. The DPP assay satisfactorily excluded diseases that may exhibit clinical presentations similar to leptospirosis and cross-reacting antibodies, establishing its suitability for the acute care setting. It also performed well in at-risk Brazilian blood donors. Specificity was relatively low in samples from healthy residents of a slum population highly exposed to *Leptospira* [5] compared with that for samples from residents of the same slum that presented to clinic with acute febrile illnesses. We included sera from healthy slum residents without regard to MAT status, whereas sera from residents with acute fever were screened for negative MAT titers in both the acute and convalescent phases. These observations suggest that the presence of low-level agglutinating antibody titers, which may persist for months to years [4], [46], even after mild disease [47], affected test performance in this group.

In the hospital setting, a positive DPP result predicted disease status with high probability. However, a negative result did not effectively exclude leptospirosis among severe disease suspects. Further diagnostic evaluation for leptospirosis should be pursued in hospitalized patients with high suspicion for leptospirosis, particularly at early stages of infection. In outpatient settings where the prevalence of leptospirosis is typically low, clinicians should use clinical and epidemiological reasoning in selecting patients for DPP testing and thereby enhance pre-test probability for leptospirosis. We showed that stratifying severe disease case-patients by end organ injury, manifested as jaundice and elevated serum creatinine, correlated with DPP positivity. Even though the model was biased toward the sickest patients, these findings suggest a potential means for stratifying PPV on clinical criteria.

Ours is the first evaluation of a field-ready rapid assay for leptospirosis to stratify test performance simultaneously by both disease severity and duration of symptoms. The results suggest that performance of serological assays for leptospirosis should ideally be evaluated in the context of both. The DPP assay was developed principally for earlier diagnosis of acute clinical leptospirosis and we established its utility in that respect. We expect the assay to also provide more timely diagnostic information for public health surveillance. Nonetheless, our study has limitations. The DPP assay relies on subjective visual interpretation for diagnosis and weakly reactive assays may be ambiguous. Further, we included some case-patients in this study without paired sera and, although we conservatively confirmed them with a high single-titer MAT threshold (≥1∶800), therefore we did not observe a rise in titers in these individuals. The lack of convalescent samples may have also contributed to the wider variation in presumptive infecting serogroup for Recife cases. The referral process for specialty care of severe leptospirosis was more centralized in Salvador than in Recife during the study period, thereby possibly making Recife case-patients less representative of the regional severe leptospirosis patient population. Lastly, we defined leptospirosis cases using an imperfect gold standard test, probably resulting in an underestimate of the DPP assay's diagnostic performance [48].

In summary, the field-ready DPP assay displayed acceptable diagnostic performance for severe leptospirosis, was highly reproducible, and can be easily implemented in hospitals where leptospirosis is a major public health problem. The next generation assay must improve detection of mild and early-phase illness, and previous work suggests that increased accuracy may be achieved with independent measurement of IgM and IgG antibodies in areas of high endemic transmission [25], [49]. The results from this study may be generalizable throughout urban Brazil where the epidemiology of leptospirosis is similar [37], [50], yet the diagnostic value of the DPP assay should be evaluated in other epidemiological settings and in serial patients with clinical syndromes consistent with leptospirosis to validate its point-of-care efficacy using whole blood.

## Supporting Information

Table S1 **STARD checklist for reporting studies of diagnostic accuracy.** From: Nabity SA, Ribeiro GS, Aquino CL, et. al. Accuracy of a Dual Path Platform (DPP) Assay for the Rapid Point-of-Care Diagnosis of Human Leptospirosis. *PLoS NTD* 2012
(DOC)

Table S2 **Serogroup, serovar, and strain of reference** ***Leptospira*** **used for the microagglutination test (MAT).** ^*^ Strain isolated at the Oswaldo Cruz Foundation (FIOCRUZ) laboratory in Salvador, Brazil from a locally identified case-patient. From: Nabity SA, Ribeiro GS, Aquino CL, et. al. Accuracy of a Dual Path Platform (DPP) Assay for the Rapid Point-of-Care Diagnosis of Human Leptospirosis. *PLoS NTD* 2012.
(DOCX)

Table S3 **Characteristics of confirmed human leptospirosis cases for which acute-phase samples were evaluated.** **NOTE.** SD = standard deviation; ND = not determined; NA = not applicable; MAT = microagglutination test. ^*^ Difference between severe cases from Salvador was statistically significant (chi-square or ANOVA *P*<0.05). **^†^** Defined as reported fever prior to clinical presentation and/or measured (≥38°C) fever by clinician at presentation. ^‡^ Defined as respiratory rate ≥30 breaths per second. ^§^ Defined by the presence of hemoptysis. ^∥^ Defined as an undetectable acute-phase MAT titer followed by a convalescent-phase titer of ≥1∶200. ^¶^ Defined by the serogroup with the highest MAT titer among both acute and convalescent specimens, where available. Approximate normal values: total leukocyte count (3,800–9,800/µL); creatinine (0.5–1.2 mg/dL); total bilirubin (0.2–1.3 mg/dL); respiratory rate (12–20 breaths per second). From: Nabity SA, Ribeiro GS, Aquino CL, et. al. Accuracy of a Dual Path Platform (DPP) Assay for the Rapid Point-of-Care Diagnosis of Human Leptospirosis. *PLoS NTD* 2012.
(DOCX)
