# Supplementary material for: Accuracy of a Dual Path Platform (DPP) Assay for the Rapid Point-of-Care Diagnosis of Human Leptospirosis
Source: PLoS Negl Trop Dis. 2012 Nov 1;6(11):e1878. doi: 10.1371/journal.pntd.0001878 (PMC3486890; doi:10.1371/journal.pntd.0001878)
Supplement: Table S2 — Serogroup, serovar, and strain of reference Leptospira used for the microagglutination test (MAT). * Strain isolated at the Oswaldo Cruz Foundation (FIOCRUZ) laboratory in Salvador, Brazil from a locally identified case-patient. From: Nabity SA, Ribeiro GS, Aquino CL, et. al. Accuracy of a Dual Path Platform (DPP) Assay for the Rapid Point-of-Care Diagnosis of Human Leptospirosis. PLoS NTD 2012. (DOCX) [file pntd.0001878.s002.docx]

| **Table S2. Serogroup, serovar, and strain of reference *Leptospira* used for the microagglutination test (MAT).** | | | | | | | |
| --- | --- | --- | --- | --- | --- | --- | --- |
| **Serogroup** | **Serovar** | **Strain** | **Sample group by site** | | | | |
|  |  |  | Recife, Brazil | Salvador,  Brazil | | | |
|  |  |  | Severe Leptospirosis | Severe Leptospirosis | Healthy Slum Resident Controls | Screening Panel: Mild Leptospirosis & Febrile Outpatient Controls | Extended Battery: Mild Leptospirosis & Febrile Outpatient Controls |
| 1) Icterohaemorrhagiae | *copenhageni* | Fiocruz L1 130^*^ | X | X | X | X | X |
| 2) Icterohaemorrhagiae | *copenhageni* | M 20 | X | X | X | X | X |
| 3) Canicola | *canicola* | H. Utrecht IV | X | X | X | X | X |
| 4) Semaranga | *patoc* | Patoc 1 | X | X | X | X | X |
| 5) Autumnalis | *autumnalis* | Akiyami A | X | X | X | X | X |
| 6) Ballum | *ballum* | Mus 127 | X | X | X | X | X |
| 7) Grippotyphosa | *grippotyphosa* | Duyster | X | X | X | X | X |
| 8) Cynopteri | *cynopteri* | 3522 C | X | X |  |  | X |
| 9) Shermani | *shermani* | 1342 K | X | X |  |  | X |
| 10) Tarassovi | *tarassovi* | Perepelicin | X | X |  |  | X |
| 11) Australis | *bratislava* | Jez Bratislava | X |  |  |  | X |
| 12) Ballum | *castellonis* | Castellon 3 | X |  |  |  | X |
| 13) Bataviae | *bataviae* | Van Tienen | X |  |  |  | X |
| 14) Celledoni | *celledoni* | Celledoni | X |  |  |  | X |
| 15) Djasiman | *djasiman* | Djasiman | X |  |  |  | X |
| 16) Hebdomadis | *hebdomadis* | Hebdomadis | X |  |  |  | X |
| 17) Icterohaemorrhagiae | *icterohaemorrhagiae* | RGA | X |  |  |  | X |
| 18) Javanica | *coxi* | Cox | X |  |  |  | X |
| 19) Louisiana | *louisiana* | LSU 1945 | X |  |  |  | X |
| 20) Panama | *panama* | CZ 214 K | X |  |  |  | X |
| 21) Pomona | *pomona* | Pomona | X |  |  |  | X |
| 22) Pyrogenes | *pyrogenes* | Salinem | X |  |  |  | X |
| 23) Sejroe | *hardjo* | Hardjoprajitno | X |  |  |  | X |
| 24) Sejroe | *wolffi* | 3705 | X |  |  |  | X |
| 25) Grippotyphosa | *canalzonae* | CZ 188 | X |  |  |  | X |
| 26) Mini | *mini* | Sari | X |  |  |  | X |
