# Supplementary material for: Accuracy of a Dual Path Platform (DPP) Assay for the Rapid Point-of-Care Diagnosis of Human Leptospirosis
Source: PLoS Negl Trop Dis. 2012 Nov 1;6(11):e1878. doi: 10.1371/journal.pntd.0001878 (PMC3486890; doi:10.1371/journal.pntd.0001878)
Supplement: Table S3 — Characteristics of confirmed human leptospirosis cases for which acute-phase samples were evaluated. NOTE. SD = standard deviation; ND = not determined; NA = not applicable; MAT = microagglutination test. * Difference between severe cases from Salvador was statistically significant (chi-square or ANOVA P<0.05). † Defined as reported fever prior to clinical presentation and/or measured (≥38°C) fever by clinician at presentation. ‡ Defined as respiratory rate ≥30 breaths per second. § Defined by the presence of hemoptysis. ∥ Defined as an undetectable acute-phase MAT titer followed by a convalescent-phase titer of ≥1∶200. ¶ Defined by the serogroup with the highest MAT titer among both acute and convalescent specimens, where available. Approximate normal values: total leukocyte count (3,800–9,800/µL); creatinine (0.5–1.2 mg/dL); total bilirubin (0.2–1.3 mg/dL); respiratory rate (12–20 breaths per second). From: Nabity SA, Ribeiro GS, Aquino CL, et. al. Accuracy of a Dual Path Platform (DPP) Assay for the Rapid Point-of-Care Diagnosis of Human Leptospirosis. PLoS NTD 2012. (DOCX) [file pntd.0001878.s003.docx]

**Table S3. Characteristics of confirmed human leptospirosis cases for which acute-phase samples were evaluated.**

|  |  | **Severe cases** | | | | **Mild cases** | |
| --- | --- | --- | --- | --- | --- | --- | --- |
|  |  | **Salvador (N=259)** | | **Recife (N=23)** | | **Salvador (N=28)** | |
| **Category** | **Characteristic** | **N** | **n (%) or mean ± SD** | **N** | **n (%) or mean ± SD** | **N** | **n (%) or mean ± SD** |
| Demographics | Age | 258 | 34 ± 14 | 23 | 32 ± 15 | 28 | 27 ± 17^*^ |
|  | Male sex | 259 | 219 (85) | 23 | 19 (83) | 28 | 19 (68)^*^ |
| Clinical presentation | Days of symptoms | 259 | 8.5 ± 4.1 | 23 | 8.2 ± 2.7 | 28 | 3.1 ± 2.6^*^ |
|  | Fever**^†^** | 254 | 251 (99) | 23 | 22 (99) | 28 | 28 (100) |
|  | Jaundice | 258 | 234 (91) | 23 | 17 (74)^*^ | 27 | 3 (11)^*^ |
|  | Oliguria | 100 | 36 (36) | 23 | 2 (9)^*^ | 27 | 3 (11)^*^ |
|  | Tachypnea^‡^ | 188 | 37 (18) | 19 | 0 (0) | 20 | 1 (5) |
|  | Pulmonary hemorrhage^§^ | 229 | 44 (19) | 22 | 6 (27) | 28 | 0 (0)^*^ |
| Laboratory evaluation on identification | Total leukocyte count (10^3^/µL) | 250 | 14.5 ± 6.9 | 23 | 11.9 ± 4.6 | 9 | 10.7 ± 6.5 |
|  | Creatinine (mg/dL) | 187 | 3.7 ± 2.4 | 23 | 2.2 ± 2.6^*^ | ND |  |
|  | Bilirubin, total (mg/dL) | 122 | 17.2 ± 13.0 | 20 | 9.9 ± 11.1^*^ | ND |  |
| Clinical outcome | Days of hospitalization | 259 | 9.6 ± 9.0 | 23 | 12.5 ± 11.1 | NA |  |
|  | Admitted to intensive care | 258 | 69 (27) | 23 | 7 (30) | NA |  |
|  | Died | 259 | 19 (7) | 23 | 0 (0) | 28 | 0 (0) |
| Paired acute and convalescent samples |  | 259 | 259 (100) | 23 | 5 (22)^*^ | 28 | 26 (93) |
| MAT confirmation criteria | Seroconversion^\|\|^ | 259 | 144 (56) | 23 | 1 (4)^*^ | 28 | 12 (43) |
|  | ≥4-fold rise in titer |  | 39 (15) |  | 5 (22) |  | 3 (11) |
|  | ≥1:800 single titer |  | 76 (29) |  | 17 (74) |  | 13 (46) |
| Presumptive serogroup^¶^ | Icterohaemorrhagiae | 259 | 249 (96) | 23 | 16 (70)^*^ | 28 | 26 (93) |
